# Medicaid Unwinding Experiences in Dual-Eligible Older Adults

**DOI:** 10.1001/jamahealthforum.2024.4692

**Published:** 2025-01-10

**Authors:** Renuka Tipirneni, Wendy Furst, Dominic A. Ruggiero, Dianne C. Singer, Erica Solway, Erin Beathard, Syama R. Patel, Andrei R. Stefanescu, Jeffrey T. Kullgren, John Z. Ayanian, Eric T. Roberts

**Affiliations:** 1 Division of General Medicine, Department of Internal Medicine, University of Michigan, Ann Arbor; 2 Institute for Healthcare Policy & Innovation, University of Michigan, Ann Arbor; 3 Division of General Internal Medicine, University of Pennsylvania, Philadelphia; 4 Veterans Affairs Center for Clinical Management Research, Veterans Affairs Ann Arbor Healthcare System, Ann Arbor, Michigan; 5 Department of Health Management and Policy, School of Public Health, University of Michigan, Ann Arbor; 6 Gerald R. Ford School of Public Policy, University of Michigan, Ann Arbor; 7 Editor in Chief, *JAMA Health Forum*

## Abstract

**Importance:**

Dual-eligible older adults rely on Medicaid to pay for Medicare premiums and cost sharing in addition to supplemental services including dental and long-term care. However, the unique experiences of dual-eligible older adults with Medicaid unwinding remain unknown.

**Objective:**

To assess the awareness and experiences of dual-eligible older adults with Medicaid redetermination.

**Design, Setting, and Participants:**

A cross-sectional national survey of community-dwelling US adults aged 65 years or older with incomes less than or equal to 100% of the federal poverty level, via internet and telephone, was conducted from January 23 through February 19, 2024. Participants were recruited from NORC probability-based and 2 additional national nonprobability panels.

**Main Outcomes and Measures:**

Weighted percentage values for respondent awareness of Medicaid redeterminations, experiences navigating reenrollment, and cost-related barriers to accessing care.

**Results:**

Of 843 respondents, most were female (62.9%), aged 65 to 74 years (62.3%), and had completed up to high school education (72.3%). Overall, 16.1% (95% CI, 12.4%-19.9%) had heard a lot and 34.6% (95% CI, 28.9%-40.4%) a little about states returning to Medicaid renewals; 49.0% (95% CI, 43.0%-55.0%) heard nothing at all. A total of 45.1% completed a Medicaid renewal, 37.0% did not complete a renewal, and 17.7% did not know about renewal requirements. A total of 87.7% maintained Medicaid, 5.9% lost Medicaid but got it back, and 5.5% lost Medicaid and did not get it back. In the last 6 months, 7.7% reported delaying or forgoing care due to cost. Delayed or forgone care was more common among those who lost Medicaid and did not get it back (18.4%) and those who lost Medicaid but got it back (30.6%) compared with those who maintained Medicaid (5.5%). Cost-related barriers were more common for dental (25.1%) and home health services (18.5%), which are frequently covered by Medicaid.

**Conclusions and Relevance:**

The findings highlight a need to address informational gaps and navigational barriers related to Medicaid unwinding among older adults with dual eligibility for Medicare and Medicaid. Addressing these gaps may help to avoid Medicaid losses that contribute to difficulties accessing care.

## Key Points

**Question:**

What are the experiences of dual-eligible older adults with Medicaid redeterminations following the unwinding of continuous Medicaid coverage during the COVID-19 public health emergency?

**Findings:**

This national cross-sectional survey study of 843 older adults (age ≥65 years) with dual eligibility for Medicare and Medicaid found varying awareness of Medicaid unwinding. Those who recently lost Medicaid reported higher cost-related access barriers.

**Meaning:**

Results of this survey study suggest that ongoing assistance is needed to help dual-eligible individuals navigate the Medicaid unwinding process and close gaps in Medicaid coverage that may impede access to care.

## Teaser

This survey study assesses the awareness and experiences of Medicaid redetermination in dual-eligible older adults.

## Introduction

On April 1, 2023, US states began to unwind policies that enabled Medicaid beneficiaries to remain continuously enrolled without completing periodic eligibility redeterminations. These policies were initially implemented in response to the COVID-19 public health emergency.^1^ On unwinding of these policies, federal guidance established that states must complete all Medicaid redeterminations within 14 months. As of August 2024, more than 25 million adults and children were disenrolled from Medicaid,^2,3^ and it is estimated that approximately 2 million adults younger than 65 years were disenrolled in the first 3 months of unwinding.^4^ A recent study^5^ of non–dual-eligible adult Medicaid enrollees found low awareness of the need for Medicaid renewals and many challenges completing the process but higher rates of renewals among those who received information about renewals. Evidence highlights substantial variation in rates of successful renewals, including by enrollee characteristics such as race and ethnicity and education, and state policies, including uses of other sources of income data to verify eligibility (ie, ex parte renewals).^6,7,8^

In addition to children and working-age adults, millions of older adults and people with disabilities with low incomes who are dually eligible for Medicare and Medicaid have been expected to complete a Medicaid redetermination for the first time in up to 3 years.^9,10^ Dual-eligible older adults may find the redetermination process to be particularly challenging due to advanced age, health risks, and low socioeconomic status.^9^ For dual-eligible older adults, Medicaid provides supplemental insurance that pays for Medicare premiums and cost sharing and for many also covers long-term care (including home health care) and services such as dental care.^11,12,13^ Medicaid loss is consequential for older adults and has previously been associated with increased cost-related barriers to care,^14^ lower use of outpatient care and medications,^15^ and widened racial and ethnic disparities in care.^16,17^

For older adults and people with disabilities who rely on both Medicare and Medicaid, the speed and complexity of redeterminations raise concerns about abrupt changes in Medicaid coverage, including Medicaid loss.^18^ Several factors underlie these concerns. First, Medicaid eligibility rules for older adults and people with disabilities often require individuals to provide detailed documentation of income and assets.^19,20^ These criteria are more complex than for adults who qualify for Medicaid through the Affordable Care Act expansion, who only need to report adjusted gross income. Second, older adults navigate a complex set of Medicaid programs with different income and asset limits in each state,^21^ and small changes in income or assets may result in individuals qualifying for different—or no—Medicaid assistance. Older adults may also face unique challenges navigating changes to Medicaid benefit eligibility (eg, from full to partial Medicaid), putting them at risk for not enrolling or reenrolling in Medicaid programs for which they are eligible.^22,23,24^

Although dual-eligible older adults have to navigate these complex redeterminations and face potentially greater health consequences of Medicaid disenrollment, no research to date has focused on the unique experiences of dual-eligible older adults with Medicaid unwinding. This study assessed low-income older adults’ awareness of Medicaid redeterminations, experiences navigating redeterminations, and the association of Medicaid coverage loss with access to care.

## Methods

### Study Design

We conducted a cross-sectional national survey of older adults with incomes less than or equal to 100% of the federal poverty level (FPL) during January 23 through February 19, 2024, as Medicare beneficiaries with incomes less than or equal to 100% FPL and low assets qualify for Medicaid supplemental coverage. Full Medicaid supplemental covers all Medicare premiums, assistance with Medicare out-of-pocket costs, long-term care, and dental care, depending on the state, while partial Medicaid assists with Medicare premiums, and in some cases, cost sharing.^25^ Among states reporting data on Medicaid renewals by age group, states reviewed Medicaid eligibility for 17% to 79% of older adults by the time of our survey’s fielding (eMethods 1 in Supplement 1). The University of Michigan institutional review board reviewed the study and determined it was exempt because it only involved interaction with participants via surveys. The study followed the American Association for Public Opinion Research (AAPOR) reporting guideline.

### Survey Sampling and Administration

Our survey included adults aged 65 years or older with Medicare coverage who self-identified as Black, Hispanic, or non-Hispanic White. Black and Hispanic adults were oversampled. NORC at the University of Chicago administered the survey to a combined probability-based panel (AmeriSpeak) designed to be representative of the US population and calibrated with 2 additional national nonprobability panels, which were used to sample sufficient respondents across sociodemographic strata.^26^ Written informed consent was obtained from probability and nonprobability participants during initial enrollment into panels. eMethods 2 in Supplement 1 presents additional details.

The survey, offered in English and Spanish, was administered online and by phone to AmeriSpeak respondents and online only to nonprobability respondents. Screener questions were administered to determine whether respondents had household incomes less than or equal to 100% FPL and how many months they had Medicaid coverage in the last 12 months. Individuals reporting 1 to 12 months of Medicaid in the last year were invited to participate in the Medicaid unwinding survey. There were no other inclusion criteria or stratification.

### Survey Measures

We used established survey items to assess low-income older adults’ awareness of Medicaid redeterminations, experiences navigating reenrollment, and impacts of coverage disruptions on health care access and health (eMethods 3 in Supplement 1).

Awareness included how much respondents heard about their state returning to Medicaid renewals, communication they received about needing to renew (a lot, a little, or nothing at all), and, among those who heard about renewals, how they heard about the process (eg, social media, family or friends). We also assessed respondents’ experiences with renewals, including receipt of help and problems completing renewals. Both awareness and experience-related survey items were adapted from the Urban Institute Health Reform Monitoring Survey.^27^ Impacts of coverage disruptions included whether respondents delayed or did not get health care due to cost or transportation problems. These access-related survey items were adapted from the National Health Interview Survey.^28^

We used respondent-reported race and ethnicity to define racial and ethnic groups. Two survey items assessed race and ethnicity. Hispanic ethnicity was assessed by asking respondents whether they were “of Spanish, Hispanic, or Latino descent.” Race was assessed by asking respondents to “check one or more categories below to indicate what race or races you consider yourself to be.” These 2 items were used to categorize respondents into the 3 mutually exclusive groupings of non-Hispanic Black, Hispanic, and non-Hispanic White. The survey included questions about respondent demographics, household size, and self-rated health, including physical, mental/emotional, and oral health.

### Survey Weights

Survey weights accounting for probability of selection and nonresponse were used in all analyses to yield estimates representative of the US population (eMethods 2 in Supplement 1). The margin of error for the full sample, accounting for design effect, was ± 6.45 percentage points.

### Statistical Analysis

We estimated overall awareness of Medicaid eligibility redeterminations, experiences navigating redeterminations, and self-reported access to care as weighted percentages. We used χ^2^ tests to compare responses across subgroups. First, to identify disparities in redetermination experiences and potential consequences for health care access, we stratified estimates by respondents’ self-identified race and ethnicity. Second, we compared awareness of Medicaid unwinding among respondents who completed a Medicaid renewal in the prior 12 months, did not report completing a Medicaid renewal, or did not know if they completed a renewal. Third, we examined the proportions of respondents with cost-related delayed or forgone care among those who did and did not lose Medicaid in the prior 12 months. Results were considered statistically significant at 2-sided *P* < .05. All analyses were conducted using SAS version 9.4 (SAS Institute Inc).

## Results

### Characteristics of Survey Participants

The survey completion rate for the AmeriSpeak sample was 34.4%, and the participation rate for nonprobability panels was 94.6%. Our sample included 843 respondents, most of whom were 65 to 74 years of age (62.3%), female (62.9%), and had completed up to high school education (72.3%) (eTable 1 in Supplement 1). Self-identified race and ethnicity were 25.6% non-Hispanic Black, 23.1% Hispanic, and 51.3% non-Hispanic White. Common chronic conditions reported included high blood pressure or hypertension (62.3%), high blood cholesterol levels (47.4%), and diabetes (25.9%). A total of 21.2% of respondents rated their overall health as excellent or very good, 36.1% rated their mental or emotional health as excellent or very good, and 22.2% rated their oral health as excellent or very good. A total of 38% of respondents reported a little and 22% reported a lot of limitations to their activities of daily living. Most respondents (63.7%) reported having Medicare Advantage.

### Communication and Awareness of Unwinding

Among dual-eligible older adults, awareness of Medicaid unwinding was low overall: 16.1% (95% CI, 12.4%-19.9%) of respondents reported they heard a lot and 34.6% (95% CI, 28.9%-40.4%) heard a little about their state returning to the Medicaid renewal process, while 49.0% (95% CI, 43.0%-55.0%) heard nothing at all (Table 1). The most common type of communication received was a renewal notice (37.9%), followed by a request to update or verify eligibility information (15.4%). A total of 33.8% of respondents reported not receiving any communication about needing to renew Medicaid. Of those who heard a little or a lot about their state returning to Medicaid renewals, 45.9% received a communication from a state Medicaid program or other government agency, 28.6% received a communication from a Medicaid plan, 27.1% heard through traditional or social media, and 16.0% heard through family or friends (Table 2). There were few differences in the characteristics of older adults who were aware vs not at all aware of the Medicaid unwinding (eTable 2 in Supplement 1).

**Table 1.** Communication and Help With Medicaid Unwinding Among Dual-Eligible Older Adults

Survey item^a^	No. (n = 843)	Weighted % (95% CI)
**How much have you heard about your state returning to the routine Medicaid renewal process?^b^**		
A lot	172	16.1 (12.4-19.9)
A little	278	34.6 (28.9-40.4)
Nothing at all	390	49.0 (43.0-55.0)
**Where did you hear about your state returning to the Medicaid renewal process?** ^c^ ^,^ ^d^		
Letter or communication from state Medicaid agency or another government agency	244	45.9 (38.1-53.6)
Letter or communication from a health plan	102	28.6 (19.7-37.5)
A physician’s office, clinic, or other health care professional	41	13.2 (6.6-19.7)
Television, radio, newspapers, or social media	135	27.1 (20.8-33.4)
Family or friends	53	16.0 (9.4-22.5)
Other	19	3.3 (1.5-5.2)
**What communication did you receive about renewing your Medicaid coverage?^d^**		
Letter or communication that you will need to renew your Medicaid coverage	349	37.9 (32.6-43.3)
Request that you verify or update your mailing address or other contact information	115	12.8 (8.7-16.9)
Request that you verify or update your income or other information about eligibility	145	15.4 (11.5-19.3)
Information about different ways to renew, such as by paper application, website, or phone	126	13.4 (9.8-16.9)
Information about how to get help during the renewal process	93	9.5 (6.0-12.9)
Letter or communication that I did not need to do anything to maintain my Medicaid coverage	128	13.4 (9.5-17.4)
I did not receive any communication about needing to renew my Medicaid coverage	265	33.8 (27.8-39.9)
Other	24	3.2 (1.5-4.9)
**Did you get any help completing your most recent Medicaid renewal?** ^e^ ^,^ ^d^		
State Medicaid agency representative	47	41.9 (28.2-55.5)
Medicare plan representative	35	21.1 (11.7-30.5)
A physician’s office, clinic, or other health care professional	9	3.7 (0.5-6.8)
Counselor or navigator, such as a State Health Insurance Assistance Program	10	13.9 (1.0-26.9)
Family or friends	17	15.4 (5.9-24.8)
Someone else	4	3.6 (0.0-8.2)
I am not sure who helped me	14	9.5 (3.3-15.7)

^a^ The verbatim survey items are available in eMethods 3 of Supplement 1.

^b^ Approximately 0.3% did not answer this question.

^c^ For those who responded they heard a lot or a little about their state returning to the routine Medicaid renewal process (n = 450).

^d^ Percentages sum to more than 100% because respondents could select more than one option.

^e^ For those who completed a Medicaid renewal in the last 12 months (n = 422).

**Table 2.** Variation in Medicaid Unwinding Experiences by Race and Ethnicity

Unwinding experience	Race and ethnicity, No. (weighted %)^a^				*P* value
	Total sample	Black, Non-Hispanic	Hispanic	White, Non-Hispanic	
**Completed Medicaid renewal (last 12 mo)** ^b^					
Yes	422 (45.1)	141 (46.9)	83 (37.9)	198 (47.4)	.25
No	292 (37.0)	101 (38.1)	58 (48.6)	133 (31.2)	
Do not know	126 (17.7)	35 (14.9)	20 (13.5)	71 (20.9)	
**Change in Medicaid coverage (last 6 mo)**					
Lost Medicaid, did not get back yet	34 (5.5)	NR^c^	NR^c^	NR^c^	.01^d^
Lost Medicaid, but got back	37 (5.9)	NR^c^	NR^c^	NR^c^	
No change; kept Medicaid for last 6 mo	762 (87.7)	366 (90.3)	249 (87.5)	762 (87.7)	
**How heard about state returning to the Medicaid renewal process** ^e^ ^,^ ^f^					
Letter or communication from state Medicaid agency or another government agency	244 (45.9)	78 (49.4)	40 (25.1)	126 (54.4)	.01
Letter or communication from a health plan	102 (28.6)	30 (22.5)	28 (43.4)	44 (24.5)	.13
A physician’s office, clinic, or other health care professional	41 (13.2)	17 (19.1)	10 (18.7)	14 (6.9)	.17
Television, radio, newspapers, or social media	135 (27.1)	49 (22.7)	30 (24.9)	56 (30.7)	.54
Family or friends	53 (16.0)	20 (14.8)	16 (30.9)	17 (9.0)	.01
**Received help completing most recent Medicaid renewal** ^g^ ^,^ ^b^					
Yes	119 (28.8)	43 (30.6)	33 (46.3)	43 (21.7)	.02
No	300 (70.8)	96 (68.8)	50 (53.7)	154 (78.0)	
**Experienced problems completing most recent Medicaid renewal** ^g^ ^,^ ^b^					
Yes	32 (6.8)	12 (12.8)	NR^c^	14 (4.7)	.05
No	388 (92.9)	129 (87.2)	NR^c^	183 (94.9)	

Abbreviation: NR, not reported.

^a^ Two survey items assessed race and ethnicity. Hispanic ethnicity was assessed by asking respondents whether they were “of Spanish, Hispanic, or Latino descent.” Race was assessed by asking respondents to “check one or more categories below to indicate what race or races you consider yourself to be.” These 2 items were used to categorize respondents into the 3 mutually exclusive groupings of non-Hispanic Black, Hispanic, and non-Hispanic White.

^b^ Percentages do not sum to 100% because a small proportion of respondents indicated that they did not know or did not answer the question.

^c^ Not reported due to small samples of respondents <10 in some cells.

^d^ *P* value indicates statistically significant differences within total sample by change in Medicaid coverage.

^e^ Participants were asked to select all that apply among response options, so percentages may sum to more than 100%.

^f^ Percentages sum to more than 100% because respondents could select more than one option.

^g^ For those who completed a Medicaid renewal in the last 12 months (n = 422).

### Experiences With Renewal Process

Of respondents who completed a Medicaid renewal, 28.8% reported receiving help with the renewal process (Table 2). A total of 6.8% reported experiencing problems completing their most recent Medicaid renewal. Enrollees in Medicare Advantage plans were significantly more likely to have completed a Medicaid renewal (51.7%) compared with enrollees in traditional Medicare (33.3%) (eTable 4 in Supplement 1). A total of 11.4% of respondents reported that they had lost Medicaid coverage in the last 6 months, including 5.5% who lost Medicaid and did not get it back and 5.9% who lost Medicaid but got it back, while 87.7% maintained Medicaid coverage (Table 2). Approximately 0.9% did not answer or reported not knowing whether they had lost Medicaid. Older adults who lost Medicaid for at least 1 month had lower educational attainment than those who maintained coverage over the prior 6 months (eTable 5 in Supplement 1).

### Variation in Unwinding Experiences by Race and Ethnicity

Higher proportions of Black (49.4%) and non-Hispanic White respondents (54.4%) heard about their state returning to the Medicaid renewal process through a letter or communication from their state Medicaid agency compared with Hispanic respondents (25.1%) (Table 2). Significantly more Hispanic respondents heard about the renewal process from family or friends (30.9%) compared with Black (14.8%) and non-Hispanic White (9.0%) respondents. Overall, 28.8% of respondents received help completing a Medicaid renewal, and 30.6% of Black, 46.3% of Hispanic, and 21.7% of non- Hispanic White respondents received help with renewals. In addition, more Black respondents (12.8%) experienced problems completing the most recent Medicaid renewal compared with non-Hispanic White (4.7%) respondents.

### Association of Awareness of Unwinding With Medicaid Renewals

**Figure 1.**
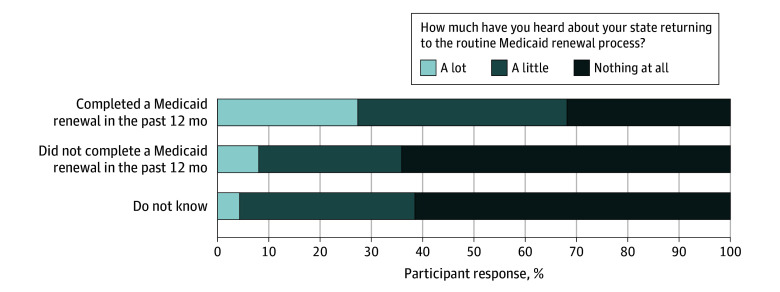
Differences in Older Adults’ Rates of Completing a Medicaid Renewal by Awareness of Medicaid Unwinding

At the time of the survey, fewer than half of respondents (45.1%) completed a Medicaid renewal in the last 12 months (Table 2). Among those who completed a renewal, 68.3% reported having heard a little or a lot about the unwinding process (Figure 1). Conversely, among respondents who did not complete or were unsure about completing a renewal, 63.2% reported having heard nothing about the unwinding process (Figure 1).

### Association of Delayed or Forgone Care With Unwinding Pathway

**Figure 2.**
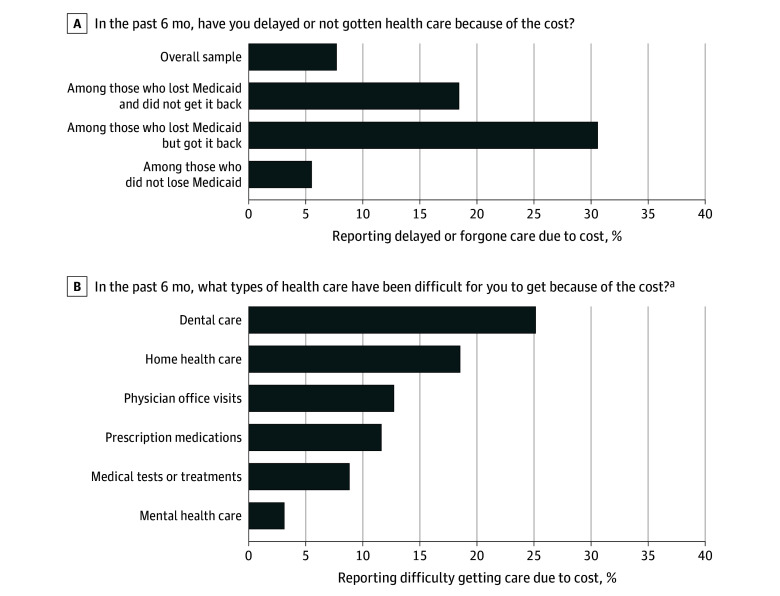
Delayed or Forgone Care Due to Cost by Pathways of Medicaid Unwinding ^a^eTable 3 in Supplement 1 presents stratified findings by Medicaid unwinding pathway (an exploratory analysis, due to small samples).

Respondents who lost Medicaid were more likely to report delaying or forgoing care due to cost concerns (Figure 2). Delayed or forgone care was reported more frequently by those who lost Medicaid and did not get it back (18.4%) and those who lost Medicaid but got it back (30.6%) compared with those who maintained Medicaid coverage (5.5%). Among older adults who reported cost-related barriers, barriers to the following types of care were reported: dental (25.1%), home health (18.5%), physician visits (12.7%), prescription medications (12.0%), medical tests or treatments (8.8%), and mental health (3.1%). We also conducted an exploratory analysis comparing differences in access and health by whether older adults lost or kept Medicaid supplemental coverage, though were limited by small samples among subgroups (eTable 6 in Supplement 1).

## Discussion

This US national survey of low-income older adults found varying awareness of and experiences with the Medicaid unwinding process and identified cost-related access barriers among those who recently lost Medicaid. By 9 to 10 months into the unwinding process, few older adults reported hearing a lot about Medicaid unwinding and many reported not receiving any communication about needing to renew Medicaid eligibility. By the time of our survey, slightly less than half of respondents had completed a Medicaid renewal. Although the proportion of respondents who said they lost Medicaid in the last 6 months was relatively low, those who lost Medicaid were more likely to report cost-related difficulty getting care. These findings highlight the importance of addressing informational and navigational barriers among dual-eligible older adults to avoid disruptions in Medicaid coverage that may contribute to difficulties accessing care.

These results add to emerging evidence of informational gaps among Medicaid enrollees during the unwinding. A KFF survey of adults aged 18 years and older who had Medicaid before the unwinding found that one-half heard little or nothing about Medicaid renewals; those who did not receive information about renewals were nearly 50 percentage points less likely to have completed a renewal than those who received information.^5^ We found similarly low rates of awareness of Medicaid unwinding among older adults. We also found that older adults who did not complete a Medicaid renewal were twice as likely to have heard nothing about the renewal process than those who completed a renewal.

Notably, among older adults who completed a Medicaid renewal by the time of our survey, only 7% experienced a problem with the process. In contrast, among respondents to the KFF survey who said they had taken action to complete a Medicaid renewal, 58% reported a problem. The lower rate of problems reported by older adults provides some indication of the success of Medicaid renewals in this population.

Various factors could have influenced the ease of completing a renewal and relatively low disenrollment rates reported by older adults. First, 35 states and Washington, DC, have existing processes for automatically verifying and renewing Medicaid eligibility for older adults and people with disabilities who receive Supplemental Security Income (SSI). These processes were linked to lower rates of Medicaid coverage loss in the prepandemic period.^29^ However, we could not identify the specific pathway by which older adults renewed Medicaid, or if disenrollment occurred less frequently in states using automatic renewal processes for dual-eligible older adults (eg, those receiving SSI). Second, approximately one-half of dual-eligible older adults are now enrolled in Medicare Advantage, and most of them are in Dual Eligible Special Needs Plans (D-SNPs).^30^ D-SNPs are specialized Medicare Advantage plans that have contracts with Medicaid programs and vary in the degree to which they coordinate and integrate Medicare and Medicaid coverage. An earlier study^17^ found that enrollment in D-SNP plans with substantial Medicaid integration was associated with a lower likelihood of Medicaid disenrollment. We found that respondents in Medicare Advantage plans were more likely to have completed a Medicaid renewal than those in traditional Medicare, but we lacked information about the type of plan individuals had or its level of Medicaid integration. Examining Medicaid disenrollment across these dimensions of coverage for dual-eligible individuals could help identify variables that may have been factors in lower Medicaid disenrollment among dual-eligible older adults compared with other Medicaid coverage groups (eg, nonelderly adults and children).^3^

Although problems with Medicaid renewals were relatively uncommon overall, we found important differences in how older adults learned about the renewal process, which could have been factors associated with variability in awareness and success of renewals. Compared with White older adults, Black and Hispanic older adults were less likely to have heard about renewal requirements through an official Medicaid communication and were more likely to have heard from family or friends. Furthermore, while Black and Hispanic adults were more likely to have received help completing a renewal than White older adults, Black older adults were more likely to have had a problem completing one. Research examining prepandemic Medicaid loss found that Black and Hispanic older adults were more likely to experience Medicaid disruptions,^17^ and a recent study of US adults (both older and nonolder) found that Black and Hispanic individuals had greater reported Medicaid loss compared with White adults.^7^ Thus, our findings underscore the continued importance of providing renewal assistance to populations that historically have been prone to such coverage disruptions, known as Medicaid churn.

Finally, our findings suggest that Medicaid coverage loss is linked to increased barriers to accessing care. While all respondents had Medicare as their primary insurance, Medicaid is an important source of supplemental coverage that helps pay for Medicare cost-sharing and services not covered by Medicare. We found that cost-related barriers to care were common among older adults, particularly for services that Medicaid primarily covers (such as dental and home health care), and that a higher proportion of individuals who lost Medicaid reported difficulty getting needed care due to cost concerns. These findings reinforce concerns about potential access challenges for those who had not undergone a Medicaid renewal by the time of our study.

### Policy implications

Our findings have several policy implications. Specifically, there is an ongoing need for outreach and assistance for older adults who lost Medicaid for procedural reasons. Although the rate of Medicaid disenrollment among dual-eligible beneficiaries was lower than that reported for other coverage groups, older adults often have greater health care needs and may be more likely to face negative consequences from increased barriers to accessing care. As many states have extended their unwinding timelines,^2,31,32^ people may only become aware that they have lost Medicaid when they attempt to use a Medicaid-covered service or have a copayment for a Medicare-covered service such as a physician visit.^5^ Further, state data suggest that states were only midway through the redetermination process by the time of our survey. Challenges could arise with the large number of renewals completed by the end of the redetermination period, and their implications might only now be surfacing. Finally, policymakers should anticipate an increase in cost-related care barriers among older adults who lose Medicaid. Help referring older adults to other forms of financial assistance for which they may qualify, including the Medicare Savings Programs (partial Medicaid benefits) and the Part D Low-Income Subsidy, could mitigate these financial barriers.

### Strengths and Limitations

Using a nationally representative survey of Medicare beneficiaries provides many strengths, including the ability to focus on a vulnerable population of low-income older adults and oversample Black and Hispanic adults. However, this study had several limitations. First, because state redetermination processes were staggered and states were at different stages of the renewal process at the time of our survey, our findings may underestimate disenrollment. Second, there is potential for recall bias if people received information about Medicaid renewals but did not remember. Third, we were unable to link respondents to administrative data to validate self-reports of Medicaid coverage and coverage loss. Prior research examining older adults found that survey-reported Medicaid enrollment broadly corresponded to administrative Medicaid enrollment data, although full and partial Medicaid may not be distinguishable.^33^ However, in the context of Medicaid enrollment growth during the COVID-19 pandemic and subsequent unwinding, it is possible that beneficiaries could have been unaware of changes in coverage status. For example, some respondents may not have known they lost Medicaid, while others may not have realized they still had Medicaid, particularly in cases of ex parte renewals (eg, when states make an automatic eligibility determination based on existing data sources, such as SSI enrollment).^8,34,35^ Fourth, compared with national characteristics of Medicare beneficiaries with low incomes before Medicaid unwinding, our survey included a smaller proportion of individuals who identified as Black or Hispanic and who had limited English proficiency (eMethods 2 in Supplement 1). Fifth, sample sizes were insufficient to examine variation in redetermination experiences across states, which may be influenced by factors such as states’ use of ex parte renewals. Lastly, our cross-sectional analyses could only descriptively characterize the association between Medicaid coverage loss and access to care.

## Conclusions

In this national survey study of older adults midway through Medicaid unwinding, awareness of unwinding was low and only half had completed a renewal, yet most kept their Medicaid supplemental coverage. Older adults who lost Medicaid coverage experienced cost-related barriers to care, which could be mitigated by greater assistance with navigating Medicaid renewals as well as referral to additional forms of financial assistance.

## Notes

Supplement 1.
eMethods 1

eMethods 2

eMethods 3
**eTable 1.** Characteristics of Survey Participants **eTable 2.** Characteristics of Older Adults Who Are Aware vs Not Aware of Medicaid Unwinding **eTable 3.** Characteristics of Older Adults Who Did vs Did Not Complete a Medicaid Renewal in the Last 12 Months **eTable 4.** Variation in Medicaid Unwinding Experiences by Insurance Type **eTable 5.** Characteristics of Older Adults Who Lost Medicaid for at Least One Month vs Did Not Lose Coverage in the Last 6 Months **eTable 6.** Differences in Access to Care and Health Status by Medicaid Unwinding Pathway
eReferences

Supplement 2.
Data Sharing Statement
