# Error in Results

**DOI:** 10.1001/jamahealthforum.2025.0120

**Published:** 2025-02-28

In the Original Investigation titled “Medicaid Unwinding Experiences in Dual-Eligible Older Adults,”^1^ which published January 10, 2025, there was a numeric error in the last paragraph of the Results. The percentage value given as “(301.6%)” should have been “(30.6%).” This article has been corrected.^1^
