# Supplementary material for: Medicaid Unwinding Experiences in Dual-Eligible Older Adults
Source: JAMA Health Forum. 2025 Jan 10;6(1):e244692. doi: 10.1001/jamahealthforum.2024.4692 (PMC11724338; doi:10.1001/jamahealthforum.2024.4692)
Supplement: Supplement 1. — eMethods 1 eMethods 2 eMethods 3 eTable 1. Characteristics of Survey Participants eTable 2. Characteristics of Older Adults Who Are Aware vs Not Aware of Medicaid Unwinding eTable 3. Characteristics of Older Adults Who Did vs Did Not Complete a Medicaid Renewal in the Last 12 Months eTable 4. Variation in Medicaid Unwinding Experiences by Insurance Type eTable 5. Characteristics of Older Adults Who Lost Medicaid for at Least One Month vs Did Not Lose Coverage in the Last 6 Months eTable 6. Differences in Access to Care and Health Status by Medicaid Unwinding Pathway eReferences [file jamahealthforum-e244692-s001.pdf]

Supplemental Online Content

Tipirneni R, Furst W, Ruggiero DA, et al. Medicaid unwinding experiences in dual-eligible older adults. *JAMA Health Forum.* 2025;6(1):e244692. doi:10.1001/jamahealthforum.2024.4692

eMethods 1

eMethods 2

eMethods 3

eTable 1. Characteristics of Survey Participants

eTable 2. Characteristics of Older Adults Who Are Aware vs Not Aware of Medicaid Unwinding

eTable 3. Characteristics of Older Adults Who Did vs Did Not Complete a Medicaid Renewal in the Last 12 Months

eTable 4. Variation in Medicaid Unwinding Experiences by Insurance Type

eTable 5. Characteristics of Older Adults Who Lost Medicaid for at Least One Month vs Did Not Lose Coverage in the Last 6 Months

eTable 6. Differences in Access to Care and Health Status by Medicaid Unwinding Pathway

eReferences

This supplemental material has been provided by the authors to give readers additional information about their work.

© 2025 Tipirneni R et al. *JAMA Health Forum.*

eMethods 1

State Medicaid Redeterminations for Older Adults as of February 2024					
**State**	**Date**	**Older Adult Redeterminations Completed, No.**	**Older Adult Medicaid Beneficiaries, No.**	**Share of Older Adults with Redeterminations Completed, %**	**Share of Total Redeterminations Completed, %**
CA	January 2024	1,060,304	1,451,232	73[a]	42
IN	February 2024	113,443	144,400	79	65
KY	February 2024	18,873	112,178	17[a]	38
MN	February 2024	43,440	96,016	45	55
ND	February 2024	8,223	10,698	77	87
OR	March 2024	70,094	124,047	57	84
PA	November 2023	169,356	322,261	53	68[b]
TN	December 2023	85,330	162,354	53	79
VA	February 2024	58,506	87,504	67	76

SOURCE: Authors' analysis of the Kaiser Family Foundation (2024) "Medicaid Enrollment and Unwinding Tracker", and individual state Medicaid unwinding dashboards and monthly reports for the states listed above.
[a]California and Kentucky did not publish demographic data for the beginning months of unwinding process.
[b]In PA, total redeterminations completed are for February 2024.

© 2025 Tipirneni R et al. *JAMA Health Forum*.

eMethods 2

AmeriSpeak® Panel

Funded and operated by NORC at the University of Chicago, AmeriSpeak® is a probability-based panel designed to be representative of the US household population. Randomly selected US households are sampled using area probability and address-based sampling, with a known, non-zero probability of selection from the NORC National Sample Frame. These sampled households are then contacted by US mail, telephone, and field interviewers (face to face). The panel provides sample coverage of approximately 97% of the U.S. household population. Those excluded from the sample include people with P.O. Box only addresses, some addresses not listed in the USPS Delivery Sequence File, and some newly constructed dwellings.

Survey Sampling

Survey participants were recruited using NORC'S AmeriSpeak® panel, a US probability panel, and the Dynata and Cint non-probability panels. Samples were built to be representative of the US population (Black, Hispanic, and White adults 65 years or older at or below 100% of the federal poverty level); Black and Hispanic individuals were purposively oversampled and weighted after data collection to their true population proportions. Only individuals with incomes ≤100% FPL and who reported on the screener question that they had any months of Medicaid in the last 12 months were included in the unwinding survey. Using the American Association for Public Opinion Research (AAPOR)'s sample performance metrics, the survey completion rate for the AmeriSpeak sample of Black, Hispanic, and White adults 65 years or older, with incomes ≤100% FPL, and Medicaid coverage in the past year, was 34.56%. The participation rate for the sample from the non-probability panels was 94.6%. As noted by AAPOR, it is not possible to calculate response rates for non-probability samples, as the proportions of invited, completions, etc., are unknown.[1]

As a formal comparison of respondents and nonrespondents is not possible for non-probability participants, we checked for similarity of our survey sample to other US samples. Specifically, we compared the characteristics of our survey participants to a national sample of Medicare Current Beneficiary Survey participants who were community-dwelling adults with incomes ≤100% FPL and at least one month of Medicaid supplemental coverage in the last year. We found broadly similar characteristics, albeit with some differences in race/ethnicity, English proficiency, and distribution of chronic conditions:

© 2025 Tipirneni R et al. *JAMA Health Forum*.

Comparison of Medicaid Unwinding Survey Participants with Medicare Current Beneficiary Survey (MCBS) Participants, Community-Dwelling with Incomes ≤100% FPL and at least one month of Medicaid in the last year		
	**Survey Respondents (N=843)**	**2021 MCBS Respondents (N=815)**
**Characteristics**	**Weighted %**	**Weighted %**
**Age**		
65-74	62.3	58.9
75+	37.7	41.2
**Gender**		
Female	62.9	67.2
**Race/ethnicity**		
Black, Non-Hispanic	25.6	37.5
Hispanic	23.1	37.3
White, Non-Hispanic	51.3	25.2
**Education**		
Less than high school	40.3	48.8
High school or equivalent	32.0	28.6
Some college/associate degree	22.3	13.1
Bachelor's degree	3.7	7.2
Post-grad study/professional degree	1.7	2.3
**Marital Status**		
Married	14.6	18.3
Widowed	25.7	28.0
Divorced	31.2	28.2
Separated	5.7	6.3
Never married	22.9	19.2
**English Speaking**		
Very Well	80.4	47.6
Well	13.0	20.0
Not Well	2.2	15.8
Not well at all	3.6	16.6
**Chronic Conditions**		
Diabetes	25.9	40.9
Hyperlipidemia	47.4	69.7
Hypertension	62.4	75.1
**Medicare Advantage**		
Medicare Advantage	63.7	63.1

© 2025 Tipirneni R et al. *JAMA Health Forum*.

Survey Weights

NORC uses a multi-stage process for generating survey weights called TrueNorth® Calibration.[2] First, probability and nonprobability sample weights are developed separately. For the AmeriSpeak® probability sample, this includes the panel weight (reflecting the cumulative panel recruitment selection probabilities, nonresponse adjustments, and calibration to population benchmarks, both at household and individual levels) and the sample selection probability for our study. This is then adjusted for nonresponse to produce the final weight for the probability sample. For the non-probability samples, the adjustment is based on raking to the same population benchmarks used for raking the probability sample. Second, small area estimation is leveraged to model core response variables and generate raking benchmarks through a machine-learning approach called gradient-boosted tree modeling. Finally, the combined probability and nonprobability sample weights are derived by raking the samples together based on these benchmarks to create the final survey weights. The design effect for the weighted sample of 843 respondents is 3.15 and the margin of error is +/- 6.45 percentage points.

© 2025 Tipirneni R et al. *JAMA Health Forum*.

eMethods 3

[For those with income ≤100% FPL (as determined by NORC) and who select "Medicaid, also known as Medical Assistance" in response to Q3 in the main survey]

The last set of questions are related to Medicaid, also known as state Medical Assistance, which is health insurance coverage for people with limited income and resources. Medicaid can help pay for Medicare premiums and co-pays when you see the doctor or go to the hospital. Medicaid can also pay for services like nursing home care or home health care. We would like to learn whether you recently experienced changes in Medicaid coverage, your ability to get health care, and your health.

1. Over the last 12 months**,** how many months did you have Medicaid? Your best estimate is fine. (check one)[a]
    a. All 12 months
    b. 6 to 11 months
    c. 1 to 5 months
    d. I did not have Medicaid at all [If Q1 = d, skip all questions in this module]
    e. Don't know

2. People with Medicaid must renew their eligibility from time to time by showing proof of their income and resources. The requirement to renew Medicaid eligibility was paused from 2020 to early 2023 due to the COVID-19 pandemic. When the Medicaid renewal process started up again in April 2023, states began asking people to provide information to renew their Medicaid coverage.
   How much have you heard about your state returning to the routine Medicaid renewal process? (check one)[a]
    a. A lot
    b. A little
    c. Nothing at all

[If Q2 = "A lot" or "A little"]

3. Where did you hear about your state returning to the Medicaid renewal process? (select all that apply)[a]
    a. Letter or communication from state Medicaid agency or another government agency
    b. Letter or communication from a health plan
    c. A doctor's office, clinic, or other health care provider
    d. Television, radio, newspapers, or social media
    e. Family or friends
    f. Other [write-in pretest only]

4. What communication did you receive about renewing your Medicaid coverage? (check all that apply)[a]
    a. Letter or communication that you will need to renew your Medicaid coverage
    b. Request that you verify or update your mailing address or other contact information
    c. Request that you verify or update your income or other information about eligibility
    d. Information about different ways to renew, such as by paper application, website, or phone
    e. Information about how to get help during the renewal process
    f. Letter or communication that I did not need to do anything to maintain my Medicaid coverage
    g. I did not receive any communication about needing to renew my Medicaid coverage
    h. Other

© 2025 Tipirneni R et al. *JAMA Health Forum*.

5. In the last 12 months, did you complete a Medicaid renewal?
    a. Yes
    b. No
    c. Don't know

[If Q5 = "Yes"]
6. Did you get any help completing your most recent Medicaid renewal? (check one)[a]
    a. Yes
    b. No [Skip to Q10]

[If Q6 = "Yes"]
7. Who helped you to complete your Medicaid renewal? (select all that apply)[a]
    a. State Medicaid agency representative
    b. Medicare plan representative
    c. A doctor's office, clinic, or other health care provider
    d. Counselor or navigator, such as a State Health Insurance Assistance Program
    e. Family or friends
    f. Someone else [write-in pretest only]
    g. I am not sure who helped me

[If Q5 = "Yes"]
8. Did you have any problems completing your most recent Medicaid renewal? (check one)[a]
    a. Yes
    b. No

[If Q8 = "Yes"]
9. What problems did you experience completing your Medicaid renewal? (select all that apply)[a,b]
    a. I never received a letter or communication about needing to renew my Medicaid coverage
    b. I had difficulty preparing and/or submitting the needed information
    c. I did not have enough time to complete my renewal
    d. I had difficulty understanding the Medicaid eligibility rules
    e. I could not find someone to answer my questions
    f. I had difficulty getting through on the phone to the state Medicaid agency
    g. I had technical problems using the Medicaid website
    h. Something else [write-in]

10. In the last 6 months, have you had a change in Medicaid coverage? (check one)
    a. Yes, I lost my Medicaid coverage and did <u>not</u> get it back yet
    b. Yes, I lost my Medicaid coverage but got it back
    c. No, I have had no change; I kept my Medicaid coverage for the last 6 months

[If Q10 = "Yes, I lost my Medicaid coverage and did not get it back yet"]
11. What is the main reason you no longer have Medicaid? (select all that apply)[c]
    a. I moved to a new state
    b. I no longer qualify for Medicaid
    c. I tried to stay enrolled in Medicaid, but I could not complete the renewal process
    d. Some other reason (please indicate)
    e. Don't know

© 2025 Tipirneni R et al. *JAMA Health Forum*.

[If Q11 = “I no longer qualify for Medicaid”]

12. If you no longer qualified for Medicaid, were you referred to any of the following (select all that apply):
    a. A Marketplace for private plans, such as HealthCare.gov
    b. Special enrollment period for Medicare
    c. A Program that helps you save money, such as a Medicare Savings Program
    d. Something else, please specify:

[If Q10 = “Yes, I lost my Medicaid coverage and did not get it back yet” or “Yes, I lost my Medicaid coverage but got it back”]

13. You said that you lost Medicaid coverage. How did you find out about this change? (select all that apply):[d]
    a. Received a communication such as a letter from a state Medicaid agency or another government agency
    b. Tried to use a Medicaid-covered service (such as dental care or home health care) and learned that I was no longer covered by Medicaid
    c. Got an unexpected bill because I no longer had Medicaid

14. In the last 6 months, have you delayed or not gotten health care because of the cost? (check one)[e]
    a. Yes
    b. No

[If Q14 = “Yes”]

15. In the last 6 months, what types of health care have been difficult for you to get because of the cost? (select all that apply)
    a. Prescription medications
    b. Doctor’s office visits
    c. Medical tests or treatments
    d. Dental care
    e. Mental health care
    f. Home health care, including support with everyday activities in your home and community

16. In the last 6 months, have you ever missed an appointment or been unable to get health care because of problems with transportation?[f]
    a. Yes
    b. No

17. In the last 6 months, would you say your health has been:[e]
    a. Excellent
    b. Very good
    c. Good
    d. Fair
    e. Poor

18. In the last 6 months, would you say your mental and emotional health has been:[e]
    a. Excellent
    b. Very good
    c. Good

© 2025 Tipirneni R et al. *JAMA Health Forum*.

d. Fair
e. Poor

19. In the last 6 months, would you say the health of your teeth and gums has been:[e]
    a. Excellent
    b. Very good
    c. Good
    d. Fair
    e. Poor

Notes:
[a] This question has been modified based on the Urban Institute's Health Monitoring Survey.[3]
[b] This question has been modified based on a State Health Access Data and Assistance Center (SHADAC) survey.[4]
[c] This question has been modified based on the Household PULSE survey.[5]
[d] This question has been modified based on the Harvard School of Public Health Southern States Survey – 2023.[6]
[e] This question has been modified based on the National Health Interview Survey.[7]
[f] This question has been modified based on the Transportation & Health Access Quality Improvement Toolkit.[8]

© 2025 Tipirneni R et al. *JAMA Health Forum*.

eTable 1. Characteristics of Survey Participants

Variable	No. (N=843)	Weighted %
**Age**		
65-74	674	62.3
75+	169	37.7
**Gender**		
Female	581	62.9
**Race/ethnicity**		
Black, Non-Hispanic	278	25.6
Hispanic	161	23.1
White, Non-Hispanic	404	51.3
**Education**		
Less than high school	103	40.3
High school or equivalent	280	32.0
Some college/associate degree	353	22.3
Bachelor's degree	72	3.7
Post-grad study/professional degree	35	1.7
**Marital Status**		
Married	110	14.6
Widowed	164	25.7
Divorced	313	31.2
Separated	44	5.7
Never married	212	22.9
**Employment status**		
Working	57	6.0
Not working	618	72.2
Disabled	168	21.7
**Geographic region**		
Northeast	115	20.3
Midwest	190	19.5
South	372	44.4
West	166	15.9
**Chronic Conditions**		
High blood pressure/hypertension	525	62.4
Diabetes/high blood sugar	237	25.9
High blood cholesterol	408	47.4
Lung disease such as chronic bronchitis or emphysema	91	10.7
A heart attack, coronary heart disease, angina, congestive heart failure, or other heart problems	112	13.4
Stroke	60	10.5
Any emotional, nervous, or psychiatric problem	118	10.1
Arthritis or rheumatism	332	40.6
**Health Status**		
Excellent/very good	187	21.2
Good/fair/poor	656	78.8
**Mental/emotional health**		
Excellent/very good	321	36.1

© 2025 Tipirneni R et al. *JAMA Health Forum*.

Good/fair/poor	513	63.1
**Oral health**		
Excellent/very good	167	22.2
Good/fair/poor	664	76.3
**Limited Activities of Daily Living**		
Yes, a lot	164	21.9
Yes, a little	330	38.0
No	349	40.1
**Medicare Advantage**		
Yes	595	63.7
No	148	24.1
Don't know	100	12.1
**Survey Language**		
English	787	91.8
Spanish	52	7.8
Other	3	0.3
**Type of Survey**		
Web	781	88.4
Phone	62	11.6

SOURCE: Authors' analysis of national survey of low-income adults ages 65 and older, fielded during January 23-February 19, 2024.

© 2025 Tipirneni R et al. *JAMA Health Forum*.

eTable 2. Characteristics of Older Adults Who Are Aware vs Not Aware of Medicaid Unwinding				
**Variable**	**Total sample (N=843) %**	**Heard anything (N=450) %**	**Heard nothing (N=390) %**	**P-value**
**Age**				0.16
65-74	62.3	66.9	57.6	
75+	37.7	33.1	42.4	
**Gender**				0.88
Female	62.9	62.5	63.5	
**Race/ethnicity**				0.65
Black, Non-Hispanic	25.6	27.6	23.7	
Hispanic	23.1	24.4	21.9	
White, Non-Hispanic	51.3	48.0	54.4	
**Education**				0.95
Less than high school	40.3	39.5	41.3	
High school or equivalent	32.0	31.8	32.1	
Some college/associate degree	22.3	23.0	21.4	
Bachelor's degree	3.7	4.2	3.3	
Post-grad study/professional degree	1.7	1.5	1.9	
**Marital Status**				0.37
Married	14.6	16.3	12.6	
Not married	85.4	83.7	87.4	
**Employment status**				0.15
Working	6.0	4.0	8.2	
Not working	72.2	77.3	66.9	
Disabled	21.7	18.8	25.0	
**Geographic region**				0.30
Northeast	20.3	16.3	24.6	
Midwest	19.5	21.8	17.0	
South	44.4	45.2	43.4	
West	15.9	16.7	15.1	
**Chronic Conditions**				
High blood pressure/hypertension	62.3	62.0	62.7	0.90
Diabetes/high blood sugar	25.9	32.2	19.3	<0.01
High blood cholesterol	47.4	47.9	46.9	0.87
Lung disease such as chronic bronchitis or emphysema	10.7	10.2	11.1	0.77
A heart attack, coronary heart disease, angina, congestive heart failure, or other heart problems	13.4	17.9	8.9	0.01
Stroke	10.5	9.0	12.0	0.55
Any emotional, nervous, or psychiatric problem	9.9	11.3	8.5	0.95
Arthritis or rheumatism	40.7	37.7	43.8	0.30
**Health Status**				0.31

© 2025 Tipirneni R et al. *JAMA Health Forum*.

Excellent/very good	21.2	18.9	23.7	
Good/fair/poor	78.8	81.1	76.3	
**Limited Activities of Daily Living**				0.19
Yes, a lot	21.9	23.3	20.4	
Yes, a little	38.1	42.0	34.0	
No	40.0	34.6	45.6	
**Medicare Advantage**				0.83
Yes	63.7	62.5	64.9	
No	24.1	25.9	22.4	
Don't know	12.1	11.7	12.7	
**Survey Language**				0.80
English	93.4	93.9	92.9	
Spanish	6.6	6.1	7.1	

© 2025 Tipirneni R et al. *JAMA Health Forum*.

eTable 3. Characteristics of Older Adults Who Did vs Did Not Complete a Medicaid Renewal in the Last 12 Months

Variable	Total sample (N=843) %	Completed Renewal (N=422) %	Did not complete Renewal (N=292) %	P value
**Age**				0.12
65-74	62.3	67.3	56.3	
75+	37.7	32.7	43.7	
**Gender**				0.51
Female	62.9	66.8	62.1	
**Race/ethnicity**				0.14
Black, Non-Hispanic	25.6	36.7	26.4	
Hispanic	23.1	19.4	30.3	
White, Non-Hispanic	51.3	53.9	43.3	
**Education**				0.60
Less than high school	40.3	35.9	42.7	
High school or equivalent	32.0	35.7	32.8	
Some college/associate degree	22.3	23.4	19.5	
Bachelor's degree	3.7	3.2	3.9	
Post-grad study/professional degree	1.7	1.8	1.1	
**Marital Status**				0.10
Married	14.6	17.6	11.1	
Not married	85.4	82.4	88.9	
**Employment status**				0.01
Working	6.0	3.1	10.9	
Not working	72.2	78.0	61.8	
Disabled	21.7	18.8	27.3	
**Geographic region**				0.05
Northeast	20.3	16.5	30.3	
Midwest	19.5	22.4	15.5	
South	44.4	43.4	41.0	
West	15.9	17.7	13.2	
**Chronic Conditions**				
High blood pressure/hypertension	62.3	63.9	61.3	0.70
Diabetes/high blood sugar	25.9	29.4	24.2	0.31
High blood cholesterol	47.4	50.6	43.6	0.30
Lung disease such as chronic bronchitis or emphysema	10.7	12.2	10.2	0.56
A heart attack, coronary heart disease, angina, congestive heart failure, or other heart problems	13.4	19.3	8.2	<0.01
Stroke	10.5	7.8	10.4	0.59
Any emotional, nervous, or psychiatric problem	9.9	12.8	5.8	0.01
Arthritis or rheumatism	40.7	35.0	46.8	0.08

© 2025 Tipirneni R et al. *JAMA Health Forum.*

**Health Status**				0.67
Excellent/very good	21.2	21.3	23.9	
Good/fair/poor	78.8	78.7	76.1	
**Limited Activities of Daily Living**				0.99
Yes, a lot	21.9	19.3	20.1	
Yes, a little	38.1	40.2	39.4	
No	40.0	40.5	40.5	
**Medicare Advantage***				0.01
Yes	63.7	73.1	57.7	
No	24.1	17.8	35.2	
Don't know	12.1	9.1	7.1	
**Survey Language**				0.76
English	93.4	92.9	91.5	
Spanish	6.6	7.1	8.5	

© 2025 Tipirneni R et al. *JAMA Health Forum*.

eTable 4. Variation in Medicaid Unwinding Experiences by Insurance Type

	Total Sample		Medicare Advantage		Traditional Medicare		
	No.	Weighted %	No.	Weighted %	No.	Weighted %	P Value
**Completed Medicaid renewal (last 12 months)**							0.01
Yes	422	45.1	322	51.7	57	33.3	
No	292	37.0	199	33.5	67	53.9	
Do not know	126	17.7	74	14.8	23	12.3	
**Change in Medicaid coverage (last 6 months)**							0.05
Lost Medicaid	71	11.4	49	7.6	11	18.4	
No change; kept Medicaid for last 6 months	762	8.6	540	92.4	136	81.6	
**How heard about state returning to the Medicaid renewal process**							
Letter or communication from state Medicaid agency or another government agency	244	45.9	177	48.7	44	42.9	0.57
Letter or communication from a health plan	102	28.6	77	23.6	16	35.9	0.25
A doctor's office, clinic, or other health care provider	41	13.2	33	14.1	[a]	[a]	
Television, radio, newspapers, or social media	135	27.1	102	30.4	25	24.2	0.43
Family or friends	53	16.0	37	14.5	[a]	[a]	

© 2025 Tipirneni R et al. *JAMA Health Forum*.

**Received help completing most recent Medicaid renewal**[b]							0.87
Yes	119	28.8	86	28.2	16	30.0	
No	300	70.8	234	71.8	41	70.0	
**Experienced problems completing most recent Medicaid renewal**[b]							0.27
Yes	32	6.8	24	7.5	[a]	[a]	
No	388	92.9	297	92.5	53	96.1	

[a]Not reported due to small samples of respondents <10 in some cells.
[b]For those who completed a Medicaid renewal in the last 12 months (N=422).

© 2025 Tipirneni R et al. *JAMA Health Forum*.

eTable 5. Characteristics of Older Adults Who Lost Medicaid for at Least One Month vs Did Not Lose Coverage in the Last 6 Months[a]

Characteristics	Lost Medicaid at least one month (N=71) N (Weighted %)	Did not lose Medicaid (N=762) N (Weighted %)	P value
**Age**			0.17
65-74	55 (47.8)	614 (64.3)	
75+	16 (52.2)	148 (35.7)	
**Gender**			0.14
Female	44 (47.0)	531 (64.9)	
**Race/ethnicity**			0.50
Black, Non-Hispanic	28 (27.4)	249 (25.6)	
Hispanic	11 (32.8)	147 (21.6)	
White, Non-Hispanic	32 (39.8)	366 (52.8)	
**Education**			0.01
Less than high school	11 (60.1)	91 (37.8)	
High school or equivalent	21 (20.1)	257 (33.6)	
Some college/associate degree	31 (16.2)	318 (23.0)	
Bachelor's degree	[b]	68 (4.0)	
Post-grad study/professional degree	[b]	28 (1.6)	
**Marital Status**			0.53
Married	12 (11.3)	95 (14.7)	
Not Married	59 (88.7)	667 (85.3)	
**Chronic Conditions**			
Diabetes	21 (14.8)	214 (27.4)	0.05
Hyperlipidemia	36 (43.8)	369 (48.1)	0.73
Hypertension	47 (61.3)	471 (62.4)	0.93
**Medicare Pathway**			0.05
Medicare Advantage	49 (52.0)	540 (74.8)	

[a]Not shown are responses for 10 individuals who responded "don't know" or did not answer the question about change in Medicaid coverage.

[b]Not reported due to small samples of respondents <10 in some cells.

© 2025 Tipirneni R et al. *JAMA Health Forum*.

eTable 6. Differences in Access to Care and Health Status by Medicaid Unwinding Pathway[a]

	Lost Medicaid/ Did Not Get Back		Lost Medicaid/ Got Back		Did Not Lose	
	No.	Weighted %	No.	Weighted %	No.	Weighted %
**Types of delayed/forgone care**						
Prescription medications	10	16.7	9	9.7	88	11.4
Doctor's office visits	6	13.1	4	31.4	68	11.5
Medical tests or treatments	5	6.0	6	33.9	61	7.3
Dental care	12	40.2	14	47.0	222	22.7
Mental health care	6	10.8	1	1.0	31	2.7
Home health care, incl support w/everyday activities	4	22.3	6	32.3	117	17.5
Don't know	1	2.4	0	0.0	29	7.4
**Missed appointment/transportation need**						
Yes	7	13.3	15	40.3	146	17.7
No	27	86.7	22	59.7	615	82.2
**Physical health**						
Excellent/very good	10	21.1	8	11.6	167	22.0
Good/fair/poor	24	78.9	29	83.8	595	78.0
**Mental/emotional health**						
Excellent/very good	13	23.7	9	13.1	295	38.6
Good/fair/poor	21	76.3	28	87.0	458	60.5
**Oral health**						
Excellent/very good	10	34.6	5	7.6	151	22.6
Good/fair/poor	23	64.1	31	91.7	602	75.9

[a]This is an exploratory analysis, due to small samples.

© 2025 Tipirneni R et al. *JAMA Health Forum.*

eReferences

1. American Association for Public Opinion Research. Standard definitions: final dispositions of case codes and outcome rates for surveys. https://aapor.org/wp-content/uploads/2024/03/Standards-Definitions-10th-edition.pdf. Accessed June 15, 2024.
2. National Opinion Research Center. NORC's Truenorth calibration tool for probability and nonprobability samples: new version 2.0 even more effective. https://truenorth.norc.org/content/dam/amerispeak/research/pdf/NORC%20-%20White%20Paper%20-%20TrueNorth%20Calibration%202.0%20-%20July%202023%20.pdf. Accessed May 10, 2024.
3. Urban Institute. Health Reform Monitoring Survey. https://www.urban.org/policy-centers/health-policy-center/projects/health-reform-monitoring-survey. Accessed May 15, 2024.
4. State Health Access Data Assistance Center [Internet]. Available from: https://www.shadac.org/
5. US Census Bureau. Household PULSE Survey. https://www.census.gov/programs-surveys/household-pulse-survey.html. Accessed June 18, 2024.
6. McIntyre A, Figueroa J, Sommers BD. Harvard School of Public Health Southern States Survey – 2023.

© 2025 Tipirneni R et al. *JAMA Health Forum.*

7. Centers for Disease Control and Prevention (CDC), National Center for Health Statistics (NCHS). National Health Interview Survey (NHIS). https://www.cdc.gov/nchs/nhis/nhis_questionnaires.htm. Accessed Sept 4, 2023.
8. Health Outreach Partners. Transportation & Health Access: a quality improvement toolkit. https://outreach-partners.org/clientportal/wp-content/uploads/2017/02/Transportation-QI-Toolkit.pdf. Accessed June 18, 2024.

© 2025 Tipirneni R et al. *JAMA Health Forum*.
