# Supplementary material for: Medicaid Unwinding Experiences in Dual-Eligible Older Adults
Source: JAMA Health Forum. 2025 Jan 10;6(1):e244692. doi: 10.1001/jamahealthforum.2024.4692 (PMC11724338; doi:10.1001/jamahealthforum.2024.4692)
Supplement: Supplement 2. — Data Sharing Statement [file jamahealthforum-e244692-s002.pdf]

Data Sharing Statement

Tipirneni. Medicaid Unwinding Experiences in Dual-Eligible Older Adults. *JAMA Health Forum*. Published January 10, 2025. doi:10.1001/jamahealthforum.2024.4692

Data
Data available: No

Additional Information
Explanation for why data not available: Aggregated data from this study available upon request from the authors.
